# Intergenerational Trauma Transmission? Test of Cellular Aging in Mothers Exposed to Sexual Abuse and Their Children

**DOI:** 10.1093/geroni/igaa057.468

**Published:** 2020-12-16

**Authors:** Laura Etzel, Waylon Hastings, Brooke Mattern, Christine Heim, Jennie Noll, Frank Putnam, Monica Oxford, Idan Shalev

**Affiliations:** 1 Penn State University, University Park, Pennsylvania, United States; 2 Pennsylvania State University, University Park, Pennsylvania, United States; 3 Charite Universitatsmedizin Berlin, Berlin, Germany; 4 University of North Carolina, Chapel Hill, North Carolina, United States; 5 University of Washington, Seattle, Washington, United States

## Abstract

Exposure to maltreatment during childhood can lead to increased risk for poor health outcomes in adulthood. Child maltreatment and later poor health may be linked by premature biological aging. We tested whether childhood sexual abuse (CSA) is associated with telomere length (TL) in adult females. We further tested the hypothesis of intergenerational transmission of trauma by measuring TL in both CSA-exposed and non-exposed mothers and their children. TL was measured in a subset of participants and their children from a prospective-longitudinal cohort study of sexually abused females and a demographically matched comparison group. Linear regression models were used to test for associations between CSA-exposure and age-adjusted TL in females (N=108, mean age 36.3 years). Multilevel linear models were used to test the intergenerational effect of maternal-CSA exposure on age-adjusted TL in their children (N=124 children mean age 10.5 years across 61 mothers). CSA-exposure was not associated with TL in females. Replicating previous work in this area, maternal TL and sex were significant predictors of child TL in all models tested. Longer maternal TL predicted longer TL in children, and female children had longer TL than male children. Maternal-CSA exposure did not predict TL in children. This finding is in line with some previous results on CSA and TL measured in adulthood. Previous significant results associating child maltreatment with shorter TL in adulthood may be capturing a population of individuals exposed to either multiple types of maltreatment or maltreatment in childhood with concurrent TL measurements.

